# Comparison of automated and manual protocols for magnetic resonance imaging assessment of liver iron concentration

**DOI:** 10.1590/0100-3984.2019.0029

**Published:** 2020

**Authors:** Izabella de Campos Carvalho Lopes, Manuel Schütze, Marina Borges Bolina, Tarcísio Ângelo de Oliveira Sobrinho, Laura Filgueiras Mourão Ramos, Renata Lopes Furletti Caldeira Diniz, Juliano de Lara Fernandes, Maria Helena Albernaz Siqueira

**Affiliations:** 1 Radiology and Diagnostic Imaging, Hospital Mater Dei, Pós-Graduação Ciências Médicas de Minas Gerais (PGCM-MG), Belo Horizonte, MG, Brazil.; 2 Cardiovascular Imaging Center, Jose Michel Kalaf Research Institute, Campinas, SP, Brazil.

**Keywords:** Liver/diagnostic imaging, Liver/metabolism, Iron overload/diagnostic imaging, Iron/metabolism, Magnetic resonance imaging/methods, Image processing, computer-assisted/methods, Fígado/diagnóstico por imagem, Fígado/metabolismo, Sobrecarga de ferro/diagnóstico por imagem, Ferro/metabolismo, Ressonância magnética/métodos, Processamento de imagem assistida por computador/métodos

## Abstract

**Objective:**

To compare automated and manual magnetic resonance imaging protocols for estimating liver iron concentrations at 1.5 T.

**Materials and Methods:**

Magnetic resonance imaging examination of the liver was performed in 53 patients with clinically suspected hepatic iron overload and in 21 control subjects. Liver iron concentrations were then estimated by two examiners who were blinded to the groups. The examiners employed automated T2* and T1 mapping, as well as manual T2* and signal-intensity-ratio method. We analyzed accuracy by using ROC curves. Interobserver and intraobserver agreement were analyzed by calculating two-way intraclass correlation coefficients.

**Results:**

The area under the ROC curve (to discriminate between patients and controls) was 0.912 for automated T2* mapping, 0.934 for the signal-intensity-ratio method, 0.908 for manual T2*, and 0.80 for T1 mapping, the last method differing significantly from the other three. The level of interobserver and intraobserver agreement was good (intraclass correlation coefficient, 0.938-0.998; *p* < 0.05). Correlations involving T1 mapping, although still significant, were lower.

**Conclusion:**

At 1.5 T, T2* mapping is a rapid tool that shows promise for the diagnosis of liver iron overload, whereas T1 mapping shows less accuracy. The performance of T1 mapping is poorer than is that of T2* methods.

## Abstract (pt)

**Objetivo:**

Comparar protocolos automatizados e manuais de ressonância magnética para estimar a concentração hepática de ferro em 1,5 T.

**Materiais e Métodos:**

Foi realizada ressonância magnética hepática em 53 pacientes com suspeita de sobrecarga de ferro hepática e 21 controles, seguida da estimativa cega da concentração hepática de ferro por dois examinadores usando mapas automáticos T2* e T1, assim como o manual T2* e o método *signal-intensity-ratio*. O desempenho foi medido usando curvas ROC e a correlação interobservador e intraobservador usando o coeficiente de correlação intraclasse bidirecional.

**Resultados:**

O desempenho da curva ROC separando pacientes e controles mostrou áreas sob a curva de 0,912 para o mapa automático T2*, 0,934 para o método *signal-intensity-ratio*, 0,908 para manual T2* e 0,80 para mapa T1 (este difere significativamente dos outros três métodos). Houve boa correlação interobservador e intraobservador (coeficiente de correlação intraclasse entre 0,938 e 0,998; *p* < 0,05). Correlações envolvendo o mapa T1, embora ainda significativas, foram menores.

**Conclusão:**

Em 1,5 T, o mapa T2* representa uma nova ferramenta rápida e promissora para avaliar o diagnóstico de sobrecarga de ferro hepática, enquanto o mapa T1 mostrou menor precisão. O desempenho do mapa T1 foi menor que o dos métodos T2*.

## INTRODUCTION

Iron overload usually results from chronic blood transfusion therapy for any one of several types of anemia^(1-3)^ or disorders of iron absorption such as hereditary hemochromatosis^(2,4)^.

Because the liver accounts for more than 70% of somatic iron storage^(1,2)^, the liver iron concentration (LIC) is an excellent surrogate marker for the whole-body iron load^(1,3,5)^, as well as helping predict the risk of intrahepatic and extrahepatic complications^(3)^.

Biochemical analysis of a liver biopsy is the gold standard for the measurement of LIC^(3-8)^ and is expressed as either mg/g liver (dry weight), with a maximum normal value of 2 mg/g, or as µmol/g, with a maximum normal value of 36 µmol/g^(9)^. However, because liver biopsy is an invasive method, magnetic resonance imaging (MRI) is chosen as the method for LIC measurement with ever-increasing frequency^(3-9)^. MRI emerged as the dominant modality for LIC measurement because of its sensitivity, reproducibility, availability, and ability to evaluate multiple organs of the body during a single imaging session^(3,5,7,10)^. MRI quantifies iron indirectly by detecting the paramagnetic effects of iron stores in the forms of ferritin and hemosiderin, interacting with nearby hydrogen nuclei. A number of empirical MRI methods for the determination of tissue iron overload have been proposed^(6)^. This has been traditionally done using manual methods, which are well-established and are validated and calibrated against the gold standard of liver biopsy, but are more user dependent and take more time to perform. More recently though, automatic methods have been proposed, with the advantage of speed and ease of use. The aim of this study was to compare automated and manual MRI protocols for estimating LIC at 1.5 T.

## MATERIALS AND METHODS

### Study design and population

This was a prospective single-center study of patients who were referred to our department for liver MRI because of suspected hepatic iron overload. We included patients with an elevated serum ferritin level (> 300 µg/L in male patients and > 200 µg/L in female patients) or elevated transferrin saturation (> 45% in male patients and > 50% in female patients). Thus, 60 eligible patients were identified. Subjects with the following conditions were excluded from the study: claustrophobia (n = 2); inability to consent (n = 1); and liver nodules detected on MRI (n = 4). Therefore, the final study group comprised 53 patients. We also evaluated 21 healthy controls, selected by convenience, with no history of liver or metabolic disease. All examinations were performed between October 2014 and August 2017. Age, gender, and body mass index were recorded for patients and controls. The study was approved by the local research ethics committee, and all participants gave written informed consent.

### MRI techniques

Unenhanced images were acquired in a 1.5 T scanner (Magnetom Avanto; Siemens Healthcare, Erlangen, Germany), with a body coil and dedicated software, version *syngo* MR B17. For the signal-intensity-ratio method proposed by the University of Rennes (URennes), we acquired five axial gradient-echo (GRE) sequences of the liver with a repetition time (TR) of 120 ms. A typical T1-weighted sequence-with an in-phase echo time (TE) of 4 ms and a flip angle of 90°-was followed by four sequences with a standard flip angle of 20° and progressively increasing T2-weighting-in-phase TE increasing from 4 to 21 ms^(4,6,11,12)^. For the manual T2* protocol, a multi-echo GRE (mGRE) sequence was used in order to acquire 12 images with increasing TE (range, 1.3-16.9 ms, with 1.4 ms intervals), a TR of 200 ms, a flip angle of 20°, a field of view of 400 × 400 mm^2^, a matrix of 96-128 × 64-96, and a slice thickness of 10 mm^(10)^.

Automated T2* and T1 maps were generated by in-line processing prototype software, the same used in the All Iron Detected Multicenter Study^(13)^. Acquisition of mGRE sequences with radiofrequency spoiling was used in order to create the T2* map^(14-16)^. The T1 map was acquired by using a modified look-locker inversion recovery (MOLLI) sequence with the 5(3)3 protocol^(14,17,18)^.

### Liver MRI analysis

Data were analyzed by two radiologists (with ten and three years of experience in abdominal radiology, respectively), both of whom were blinded to the clinical status of the subjects, and the level of interobserver agreement was assessed. One of the radiologists also repeated all measurements, in order to allow intraobserver agreement to be assessed.

Four methods for estimating LIC values were evaluated in this study. The two manual methods were well-established at the time of the study design: the URennes (signal-intensity-ratio) method proposed by Gandon et al.^(11)^; and the manual T2* (relaxometry) method proposed by Hankins et al.^(10)^. Both have been validated and calibrated against the gold standard of liver biopsy and show that MRI enables good quantification of LIC^(10-12,19-25)^. The two automatic methods evaluated were the ultrafast scanning T2* and T1 mapping techniques^(13-18)^, which were proposed more recently

### URennes method

For quantification of the URennes values, we used the algorithm developed by Gandon et al.^(11)^. That algorithm uses the liver-to-muscle signal intensity ratios in five axial GRE sequences of the liver. For every sequence, we measured liver signal intensity (SI) in three operator-defined regions of interest (ROIs) of 1-3 cm^2^ in the right lobe of the liver, avoiding large vessels, biliary tracts, parenchymatous lesions, and artifacts. Muscle SI was measured in two ROIs in the right and left paraspinal muscles. The ROIs were propagated to all five images. Values were entered manually on a website developed by the authors^(12)^, which provides LIC values in µmol Fe/g.

### Manual T2* mapping

We drew freehand ROIs directly on the scanner console over source images in a homogeneous area of the right hepatic lobe, at the level of the origin of the main portal vein, avoiding blood vessels and visible bile ducts. The SI was obtained and transferred manually to the software on another computer. The mean SI in each slice with varying TEs was used in order to fit the T2* curve by using the formula *SI* = *Ke*^−T2*/TE^, with truncation of the decay curve to achieve the best results, in accordance with previous recommendations^(10)^. The TEs were manually excluded from the fit in images with high iron-mediated signal loss.

The T2* values were transformed into their reciprocal R2* (R2*[Hz] = 1,000/T2*[ms]). The LIC values (in mg Fe/g) were estimated from R2* values using the linear regression model described by Hankins et al.^(10)^.

### T2* mapping

Automated T2* maps were calculated using in-line processing prototype software and were based on the original mGRE images of the liver. The T2* values (in ms) were obtained by drawing an ROI directly onto the map generated^(13-16)^. The LIC values (in mg Fe/g) were estimated by using the linear regression model described by Hankins et al.^(10)^.

### T1 mapping

The T1 map was measured in six circular ROIs larger than 1 cm^2^ (range, 1-3 cm^2^). We placed four ROIs in the right lobe and two in the left lobe, avoiding artifacts, major vascular structures, and lesions. The prototype software generated a native image with multiple echoes, as well as final T1 maps that had already been processed automatically^(14,17,18)^. In our calculations, we used the arithmetic mean of those ROIs. The T1 values were transformed into their reciprocal R1 values (R1[Hz] = 1000/T1[ms]), which increase during iron overload. It was not possible to estimate LIC values based on R1, because there is as yet no conversion formula for those two measures.

### Statistical analysis

For the calculation of descriptive statistics, numerical variables were submitted to the Shapiro-Wilk test of normality. Those variables are presented as mean ± standard deviation (SD) when normally distributed and as median (interquartile range) when not. Categorical variables are presented as frequencies and percentages. We compared patients and control subjects using the t-test for normally distributed variables or the Mann-Whitney-Wilcoxon test for those not so distributed. Categorical values were compared by the chi-square test.

Measures obtained with the URennes, manual T2*, automated T2* mapping, and T1 mapping protocols were compared by constructing receiver operating characteristic (ROC) curves, and the performance of the protocols was assessed by calculating the area under the curve (AUC) and the corresponding 95% confidence interval (95% CI). Correlations between measures were quantified by calculating Spearman’s correlation coefficient. The intraclass correlation coefficient (ICC) was calculated in order to determine the level of interobserver and intraobserver agreement^(26)^, and the Student’s t-test for independent samples (difference equal to zero) was used for the analysis of differences between means. Statistical analyses were performed with R software, version 3.3.2 (R Foundation for Statistical Computing, Vienna, Austria). Values of *p* < 0.05 were considered statistically significant.

## RESULTS

### Sample characteristics

In this study, we assessed LICs in 74 individuals (53 patients and 21 controls). The subject characteristics are summarized in Table 1. Patients did not differ significantly from healthy control subjects with respect to gender, age, height, or the presence of hepatic steatosis. However, the control subjects had lower body weights (*p* < 0.001). Among the patients, the mean serum ferritin was 974 µg/L in the males and 1542 µg/L in the females.

**Table 1:** Characteristics of the subjects evaluated.

		Group		
Characteristic	All subjects (n = 74)	Patients (n = 53)	Controls (n = 21)	*P*-value
Male gender, n (%)	35 (47.3)	23 (43.4)	12 (57.1)	0.418*
Age (years), mean ± SD	36.5 ± 29	33 ± 30	41 ± 36	0.084^†^
Weight (kg), mean ± SD	67.6 ± 25.3	62.0 ± 25.7	81.6 ± 17.9	< 0.001^‡^
Height (m), mean ± SD	165.5 ± 15	165 ± 20	170 ± 8	0.096^†^
Steatosis, n (%)	18 (24.3)	12 (22.6)	6 (28.6)	0.814*
Diagnoses, n^§^ (%)				
Sick-cell disease	28 (37.8)	28 (52.8)	-	-
Hemochromatosis	11 (14.9)	11 (20.8)	-	-
Hyperferritinemia	10 (13.5)	10 (18.9)	-	-
Thalassemia major	3 (4.1)	3 (5.7)	-	-
Thalassemia intermedia	3 (4.1)	3 (5.7)	-	-
Thalassemia minor	1 (1.4)	1 (1.9)	-	-

* Chi-square test of independence.

† Mann-Whitney-Wilcoxon test-data as median (interquartile range).

‡ Student’s t-test for independent samples-data as mean ± SD.

§ Two patients had sickle-cell disease and hemochromatosis, and one patient had sickle-cell disease and thalassemia major.

### Reliability of the measures

The interobserver analysis showed no significant difference between the two radiologists, with excellent agreement between the measurements (Table 2). Because the agreement between the two raters for all methods was high, correlation and ROC curve analyses were performed using the measures obtained by the first rater. The intraobserver analysis showed no significant difference between repeated measures by the same radiologist for all of the methods, except for T1 mapping (*p* < 0.05), despite the fact that the ICC for agreement between the two raters was above 0.9 for all of the protocols (Table 2).

**Table 2:** Interobserver and intraobserver agreement for all of the methods evaluated.

			Agreement		
	Interobserver			Intraobserver	
Protocol	Mean difference (95% CI)	ICC (95% CI)		Mean difference (95% CI)	ICC (95% CI)
URennes	-0.012 (-0.115 to 0.090)*	0.998 (0.996 to 0.999)^†^		0.057 (-0.240 to 0.353)*	0.986 (0.976 to 0.992)^†^
T2* mapping	0.016 (-0.142 to 0.174)*	0.980 (0.969 to 0.988)^†^		0.082 (-0.041 to 0.206)*	0.987 (0.980 to 0.992)^†^
T1 mapping	-3.878 (-10.745 to 2.988)*	0.964 (0.944 to 0.977)^†^		-10.095 (-19.170 to -1.019)^†^	0.938 (0.903 to 0.960)^†^
Manual T2*	0.007 (-0.227 to 0.241)*	0.954 (0.927 to 0.970)^†^		0.009 (-0.215 to 0.233)*	0.957 (0.933 to 0.973)^†^

* *P* > 0.05 by Student’s t-test for independent samples (difference equal to zero).

† *P* < 0.05.

### Performance of the protocols

**Figure 1:**
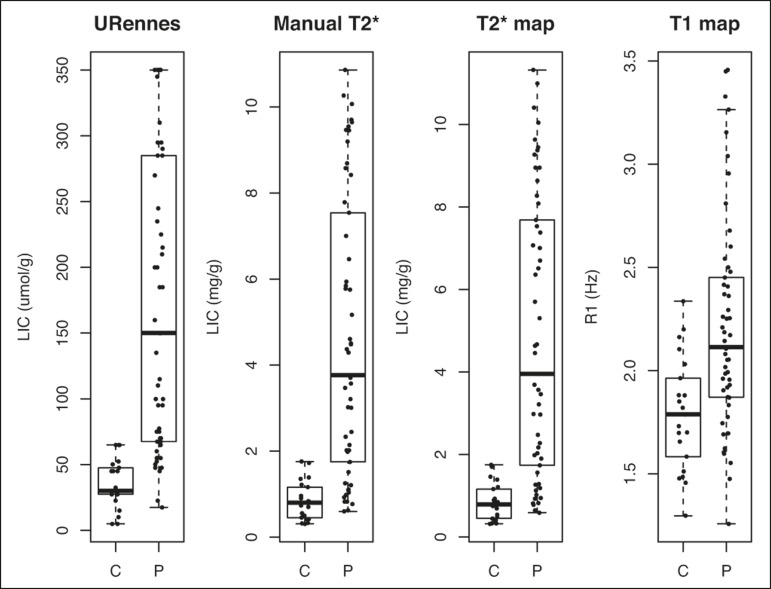
Values obtained using the URennes, manual T2*, T2* mapping, and T1 mapping protocols for patients and controls. C, controls; P, patients.

**Figure 2:**
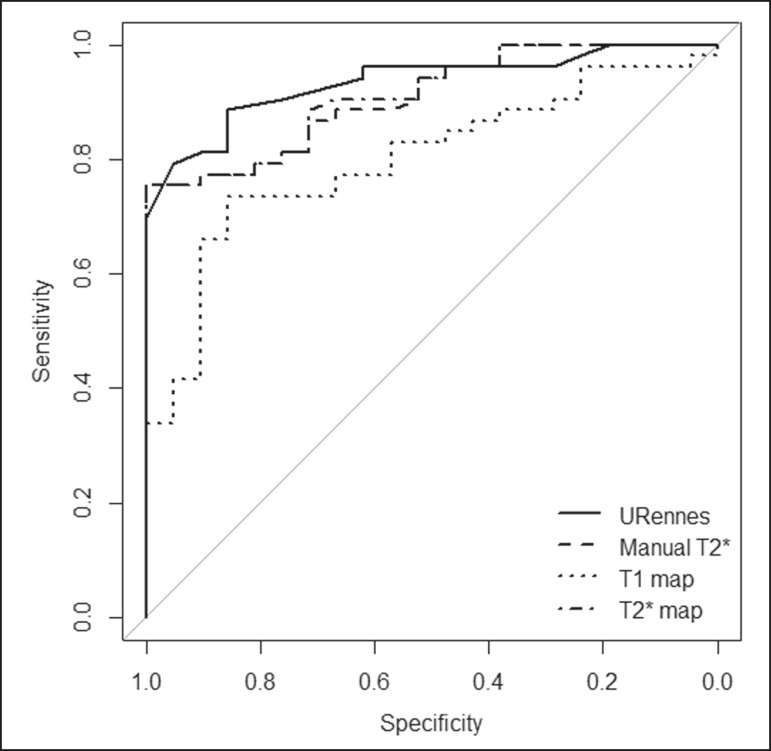
ROC curves using the URennes, manual T2*, T2* mapping, and T1 mapping protocols for separating patients and controls. The AUC (95% CI) was 0.934 (0.881-0.988) for URennes; 0.908 (0.844-0.972) for manual T2*; 0.80 (0.70-0.904) for T1 mapping; and 0.912 (0.849-0.974) for T2* mapping. The T1 mapping curve differs significantly from those of URennes (*p* = 0.008), manual T2* (*p* = 0.038), and T2* mapping (*p* = 0.031).

The scan times and average image analysis durations are listed in Table 3. Values measured by each protocol, for patients and controls, are shown in Figure 1. The URennes and manual T2* protocols showed good power of discrimination between patients and controls (AUC > 0.9). The T2* mapping protocol also had an AUC above 0.9, although the T1 mapping protocol performed poorly, with an AUC of only 0.8 (95% CI: 70-90.4%), and differed significantly from the other protocols: URennes (*p* = 0.008); manual T2* (*p* = 0.038); and T2* mapping (*p* = 0.031). The URennes, manual T2*, and T2* mapping protocols had similar power to detect patients with elevated ferritin or transferrin saturation and clinically suspected hepatic iron overload (Figure 2).

**Table 3:** Scan times, image analysis duration, and total time required to complete each of the MRI protocols evaluated.

Protocol	Scan time	Image analysis duration (mean ± SD)	Average total protocol time
URennes	109 s	423 ± 48 s	530 s
Manual T2*	11 s^†^	308 ± 39 s	320 s
T2* mapping	11 s^†^	20 ± 4 s	30 s
T1 mapping	5 s	90 ± 15 s	100 s

† Time for joint acquisition of the T2* mapping and manual T2*.

### Correlations among LIC measures obtained by the URennes, T2* mapping, and manual T2* protocols

**Figure 3:**
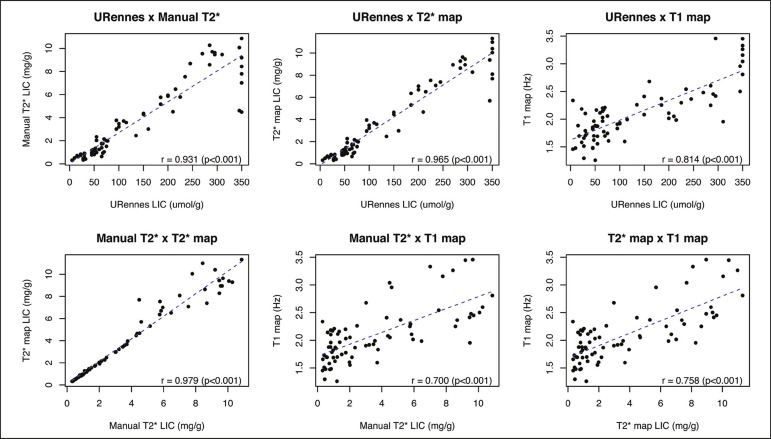
Correlations among the protocols. The blue line represents the fitted linear model. r, Spearman’s correlation coefficient (*p*-values refer to the correlation coefficient).

To study the relationships among measures obtained by the URennes, T2* mapping, manual T2*, and T1 mapping protocols, we calculated the correlation coefficients. The results are shown in Figure 3. All measures showed a significant correlation among the protocols, and the correlation coefficients were above 0.9 for the URennes, T2* mapping, and manual T2* protocols, although those associations were weaker when the URennes-measured LIC was above 350 µmol Fe/g. The strongest correlation observed was between the T2* mapping-measured LICs and those measured with the T2* manual protocol (r = 0.979). Correlations involving R1 values obtained with the T1 mapping protocol, although still significant, were lower, probably due to the great dispersion of R1 values. The T1 mapping protocol correlated most strongly with the URennes protocol (r = 0.814; *p* < 0.001).

## DISCUSSION

In this study, we applied different methods of LIC estimation using 1.5 T MRI in a group of 53 patients and 21 controls and compared the results between manual and automatic methods. The main advantages of the mapping techniques are speed and ease of use. That was confirmed in our study, in which the time required for image acquisition/processing was found to be significantly shorter for the T2* mapping and T1 mapping protocols than for the URennes and manual T2* protocols.

Shortly after the conclusion of the data collection for this study in 2017, the URennes published a study proposing a new method that employs a proton density technique, involving the use of magnitude images, and has produced quite promising results^(9)^. The authors of that study showed that the URennes method employed in the present study tends to overestimate LIC values and is susceptible to potentially serious errors if coils other than an antenna coil are employed. They recommended migrating to the Digital Imaging and Communication in Medicine MRQuantIF software that uses the Alústiza algorithm by default^(21)^, which controls the acquisition parameters^(9)^. Because that software would require the re-acquisition of imaging data using a different protocol, the remainder of the discussion will focus primarily on the manual T2*, T2* mapping, and T1 mapping protocols.

In the present study, the level of interobserver agreement was good, the ICC being above 0.9 for all four protocols. The level of intraobserver agreement was also good, although the t-test showed a significant difference between measures obtained with the T1 mapping protocol. That is likely related to the higher dispersion of R1 values estimated using this method.

Native T1 mapping detects elevated levels of extracellular water, thus identifying fibrosis^(18)^, which is reflected in an increased T1 value. However, the presence of excess iron competes with this effect, reducing T1 (and conversely increasing R1). The most widely used T1 mapping sequence is based on the MOLLI technique. Described originally by Messroghli et al.^(27)^, it consists of a single-shot true fast imaging with steady-state precession sequence with acquisitions over different inversion time readouts, allowing for magnetization recovery of a few seconds after three to five readouts. The advantages of this sequence are its acquisition in only one relatively short breath-hold, the higher spatial resolution (1.6 × 2.3 × 8 mm), and the increased dynamic signal. T1 maps are generated automatically after MOLLI image acquisition, without the need for further post-processing, which accelerates image analysis. Similarly, in-line application of motion correction provides more accurate pixel-wise maps, avoiding artifacts due to respiratory motion. Torlasco et al.^(28)^ demonstrated that T1 mapping correlates better with cardiac and hepatic iron levels than does T2* mapping. Despite those advantages, we found that T1 mapping showed the lowest ICC, the weakest correlation with the other methods, and the lowest AUC for differentiating patients from controls. Therefore, although we found a significant correlation with the R1 and LIC values estimated by the other methods, there is no clear evidence that T1 mapping could consistently estimate LIC values through the use of the protocol applied in this study. Nevertheless, there are many T1 sequences other than MOLLI that could be used-including shortened MOLLI, saturation recovery single-shot acquisition, saturation-pulse prepared heart-rate-independent inversion-recovery, and inversion time scout-each with different reference values. Further studies are needed in order to examine the relationship between R1 values and LIC.

There were strong correlations among the URennes, manual T2*, and T2* mapping protocols regarding LIC estimation, and all correlations with the URennes protocol were significant, although the correlations among LIC measures were weaker when the LIC was above 15 mg Fe/g. This corroborates current knowledge that there is saturation at 1.5 T with very high iron overload, consequently, the method cannot quantify LIC values greater than 375 µmol/kg (20.9 mg/kg) and hence does not capture the entire relevant range of values^(2)^. For T2* calculations, depending on the MRI scanner gradient system, liver T2* can measure LICs only up to 40 mg/g^(29)^. That’s because GRE sequences are inaccurate at higher iron overload levels^(2,4,10,22,25)^, given that the mGRE techniques employed are intrinsically limited when detecting rapidly decaying MRI signals. Nevertheless, higher LIC values can be measured with this method, because conventional mGRE techniques only start to become degraded during the measurement of signals with T2* times ≤ 1 ms, corresponding to LIC values above approximately 25 mg Fe/g liver (dry weight) at 1.5 T^(10,25)^. In contrast, d’Assignies et al.^(5)^, in a study comparing and validating the signal-intensity-ratio method and the R2* method with liver biopsy at 3.0 T, demonstrated that the biopsy-determined LIC correlates better with that determined by the R2* method than with that determined by the signal-intensity-ratio method when the degree of iron overload is slight to moderate (< 130 µmol/g). However, in patients with a high biopsy-determined LIC (≥ 130 µmol/g), the signal-intensity-ratio method correlates better.

The strongest correlation between methods was observed for that between the automated T2* mapping and manual T2* protocols. We find that interesting, because those two methods use the same image source for calculations, as well as because the time required for image acquisition and processing is approximately ten times shorter for the automated T2* mapping protocol than for the manual T2* protocol.

In the present study, we chose to use the conversion proposed by Hankins et al.^(10)^ for the manual T2* analysis. Those authors compared R2* values to the biopsy-determined LIC, using three different MRI methods, demonstrating that the estimations obtained with the shortest first TE correlated most strongly with the directly measured values^(4,10)^. Those values are easily calculated with a Microsoft® Excel spreadsheet, on which myocardial and hepatic iron concentrations can be estimated by inputting the mean SI values for each TE.

Parametric maps require specific sequences and software capable of performing the curve fits and generating maps without the need for manual user input. Unfortunately, there is great heterogeneity among the sequences and software implemented by different MRI equipment manufacturers, resulting in variation among estimated LIC measures. This is a major limiting factor for the use of this method in clinical practice. Nevertheless, studies have shown that T2* mapping can detect liver hemosiderosis^(30)^ and can accurately identify high concentrations of iron^(7)^. In the present study, we found that T2* mapping produced results similar to those of manual T2*, with low levels intraobserver and interobserver variability.

Our study has some limitations. We employed only one 1.5 T MRI scanner, and it was therefore not possible to compare reproducibility between or among different devices. In addition, we included a limited number of subjects, especially in the control group. We also had no measure of serum ferritin in the control subjects and therefore cannot exclude the possibility of iron deposition, which can be seen in asymptomatic patients, in that group.

## CONCLUSION

Our findings indicate that T2* mapping of the liver is a promising new tool for the rapid diagnosis of hepatic iron overload, and that T1 mapping is less accurate for that purpose. Further studies are needed in order to improve understanding the value of T1 mapping in clinical practice and to propose changes to overcome intrinsic limitations of the manual T2* protocol in LIC estimation. There are various robust T2* methods that could be routinely used for estimating LIC.
